# Detailed Analysis of *ITPR1* Missense Variants Guides Diagnostics and Therapeutic Design

**DOI:** 10.1002/mds.29651

**Published:** 2023-11-14

**Authors:** Jussi Pekka Tolonen, Ricardo Parolin Schnekenberg, Simon McGowan, David Sims, Meriel McEntagart, Frances Elmslie, Debbie Shears, Helen Stewart, George K. Tofaris, Tabib Dabir, Patrick J. Morrison, Diana Johnson, Marios Hadjivassiliou, Sian Ellard, Charles Shaw‐Smith, Anna Znaczko, Abhijit Dixit, Mohnish Suri, Ajoy Sarkar, Rachel E. Harrison, Gabriela Jones, Henry Houlden, Giorgia Ceravolo, Joanna Jarvis, Jonathan Williams, Morag E. Shanks, Penny Clouston, Julia Rankin, Lubov Blumkin, Tally Lerman‐Sagie, Penina Ponger, Salmo Raskin, Katariina Granath, Johanna Uusimaa, Hector Conti, Emma McCann, Shelagh Joss, Alexander J.M. Blakes, Kay Metcalfe, Helen Kingston, Marta Bertoli, Rachel Kneen, Sally Ann Lynch, Inmaculada Martínez Albaladejo, Austen Peter Moore, Wendy D. Jones, Genomics England Research Consortium, Esther B.E. Becker, Andrea H. Németh

**Affiliations:** ^1^ Nuffield Department of Clinical Neurosciences University of Oxford Oxford UK; ^2^ Kavli Institute of Nanoscience Discovery University of Oxford Oxford UK; ^3^ Oxford Center for Genomic Medicine Oxford University Hospitals National Health Service Foundation Trust, University of Oxford Oxford UK; ^4^ Centre for Computational Biology, MRC Weatherall Institute of Molecular Medicine University of Oxford Oxford UK; ^5^ South West Regional Genetics Service St. George's University Hospitals London UK; ^6^ Northern Ireland Regional Genetics Service Belfast City Hospital Belfast UK; ^7^ Patrick G. Johnston Centre for Cancer Research and Cell Biology Queen's University Belfast Belfast UK; ^8^ Sheffield Clinical Genetics Service Sheffield Children's NHS Foundation Trust Sheffield UK; ^9^ Department of Neurology Royal Hallamshire Hospital, Sheffield Teaching Hospital NHS Foundation Trust Sheffield UK; ^10^ Exeter Genomics Laboratory Royal Devon University Healthcare NHS Foundation Trust UK; ^11^ Peninsula Clinical Genetics Service, Royal Devon University Hospital Royal Devon University Healthcare NHS Foundation Trust Exeter UK; ^12^ Department of Clinical Genetics Nottingham University Hospitals NHS Trust Nottingham UK; ^13^ Department of Neuromuscular Disorders UCL Queen Square Institute of Neurology, University College London London UK; ^14^ Unit of Pediatric Emergency, Department of Adult and Childhood Human Pathology University Hospital of Messina Messina Italy; ^15^ Birmingham Women's and Children's NHS Foundation Trust Birmingham UK; ^16^ Oxford Regional Genetics Laboratory, Churchill Hospital Oxford University Hospitals NHS Foundation Trust Oxford UK; ^17^ Department of Clinical Genetics Royal Devon and Exeter NHS Foundation Trust Exeter UK; ^18^ Sackler School of Medicine Tel Aviv University Tel Aviv Israel; ^19^ Pediatric Movement Disorders Service, Pediatric Neurology Unit Edith Wolfson Medical Center Holon Israel; ^20^ Magen Center for Rare Diseases‐Metabolic, Neurogenetic Wolfson Medical Center Holon Israel; ^21^ Movement Disorders Unit, Department of Neurology Tel Aviv Sourasky Medical Center Tel Aviv Israel; ^22^ Genetika Centro de Aconselhamento e Laboratório Curitiba Brazil; ^23^ Research Unit of Clinical Medicine Medical Research Center, Oulu University Hospital and University of Oulu Oulu Finland; ^24^ All Wales Medical Genomics Service Wrexham Maelor Hospital Wrexham UK; ^25^ Liverpool Women's Hospital Foundation Trust Liverpool UK; ^26^ West of Scotland Centre for Genomic Medicine Queen Elizabeth University Hospital Glasgow UK; ^27^ Division of Evolution, Infection and Genomics, School of Biological Sciences, Faculty of Biology Medicine and Health, University of Manchester Manchester UK; ^28^ Manchester Centre for Genomic Medicine University of Manchester, St. Mary's Hospital, Manchester Academic Health Science Centre Manchester UK; ^29^ Northern Genetics Service International Centre for Life Newcastle upon Tyne UK; ^30^ Department of Neurology Alder Hey Children's NHS Foundation Trust Liverpool UK; ^31^ Department of Clinical Genetics Children's Health Ireland (CHI) at Crumlin Dublin Ireland; ^32^ Neurology in Pediatrics Hospital Santa Lucía Murcia Spain; ^33^ The Walton Centre NHS Foundation Trust Liverpool UK; ^34^ North East Thames Regional Genetics Service Great Ormond Street Hospital for Children, Great Ormond Street NHS Foundation Trust London UK; ^35^ Department of Health & Social Care London UK

**Keywords:** ITPR1, IP_3_R1, spinocerebellar ataxia type 29, Gillespie syndrome, cerebellum, next‐generation sequencing

## Abstract

**Background:**

The *ITPR1* gene encodes the inositol 1,4,5‐trisphosphate (IP_3_) receptor type 1 (IP_3_R1), a critical player in cerebellar intracellular calcium signaling. Pathogenic missense variants in *ITPR1* cause congenital spinocerebellar ataxia type 29 (SCA29), Gillespie syndrome (GLSP), and severe pontine/cerebellar hypoplasia. The pathophysiological basis of the different phenotypes is poorly understood.

**Objectives:**

We aimed to identify novel SCA29 and GLSP cases to define core phenotypes, describe the spectrum of missense variation across *ITPR1*, standardize the *ITPR1* variant nomenclature, and investigate disease progression in relation to cerebellar atrophy.

**Methods:**

Cases were identified using next‐generation sequencing through the Deciphering Developmental Disorders study, the 100,000 Genomes project, and clinical collaborations. *ITPR1* alternative splicing in the human cerebellum was investigated by quantitative polymerase chain reaction.

**Results:**

We report the largest, multinational case series of 46 patients with 28 unique *ITPR1* missense variants. Variants clustered in functional domains of the protein, especially in the N‐terminal IP_3_‐binding domain, the carbonic anhydrase 8 (CA8)‐binding region, and the C‐terminal transmembrane channel domain. Variants outside these domains were of questionable clinical significance. Standardized transcript annotation, based on our *ITPR1* transcript expression data, greatly facilitated analysis. Genotype–phenotype associations were highly variable. Importantly, while cerebellar atrophy was common, cerebellar volume loss did not correlate with symptom progression.

**Conclusions:**

This dataset represents the largest cohort of patients with *ITPR1* missense variants, expanding the clinical spectrum of SCA29 and GLSP. Standardized transcript annotation is essential for future reporting. Our findings will aid in diagnostic interpretation in the clinic and guide selection of variants for preclinical studies. © 2023 The Authors. *Movement Disorders* published by Wiley Periodicals LLC on behalf of International Parkinson and Movement Disorder Society.

Inositol 1,4,5‐triphosphate receptors (IP_3_R1–3) are critical players in intracellular calcium signaling, mediating the release of calcium ions from the endoplasmic reticulum (ER) into the cytosol.^1^ Activation of the tetrameric channel is induced by simultaneous binding of four molecules of inositol 1,4,5‐triphosphate (IP_3_), one to each subunit comprising the IP_3_R channel.^2^, ^3^ The subunits consist of five key domains: an N‐terminal suppressor domain, the loss of which appears to increase IP_3_ binding to its binding domain^4^; the IP_3_‐binding domain; a cytosolic regulatory domain; a transmembrane channel domain; and a cytosolic C‐terminus. The domains are organized in a tetrameric “mushroom‐like” structure, with the stalk inserted into the ER membrane and the cap exposed to the cytosol.^5^ This organization makes the IP_3_ binding core accessible to IP_3_, and the regulatory domain available for many protein interactions (eg, with carbonic anhydrase 8, CAR8^6^, ^7^) and post‐translational modifications that regulate the receptor activity.^1^ Binding of IP_3_ triggers conformational changes that are transmitted intramolecularly over a large distance to open the C‐terminal channel pore.^3^, ^8^, ^9^

Expression of *ITPR1* (OMIM *147265) encoding the type 1 IP_3_R is ubiquitous, and IP_3_R1 is the predominant neuronal IP_3_ receptor enriched in the Purkinje cells of the cerebellar cortex,^10^, ^11^, ^12^, ^13^ where it regulates Purkinje cell development and calcium homeostasis.^14^, ^15^ A knockout of the *Itpr1* gene in mice results in very early lethality, severe ataxia, and epileptic seizures without an overt cellular phenotype.^13^ In humans, *ITPR1* is considered a hub gene for cerebellar ataxias.^16^ Pathogenic variants in *ITPR1* cause neurodegenerative spinocerebellar ataxia type 15 (SCA15)^17^ (OMIM #606658), and congenital SCA29^18^ (OMIM #117360), Gillespie syndrome^19^, ^20^ (OMIM #206700), and severe pontine/cerebellar hypoplasia.^21^ The IP_3_R1 channel further contributes to neurodegeneration^22^ in SCA2^23^, ^24^ (OMIM #183090), SCA3^25^ (OMIM #109150), Huntington's disease^26^, ^27^ (OMIM #143100), familial Alzheimer's disease^28^, ^29^ (OMIM #607822), and has been implicated in mouse models of *ATM‐* and *APTX*‐related ataxias,^30^ making the IP_3_R1 channel an attractive druggable target. While the enrichment of *ITPR1* expression in the Purkinje cells may account for the predominantly cerebellar features observed in *ITPR1*‐related disorders,^17^, ^18^, ^19^, ^20^, ^31^ the pathophysiological basis for these different phenotypes is poorly understood. Furthermore, cerebellar atrophy was recently highlighted as a hallmark of *ITPR1*‐related disease,^32^ but concordance between cerebellar atrophy and symptom progression is unclear.

With increasing interest in disease‐specific American College of Medical Genetics and Genomics/American Association of Molecular Pathology (ACMG/AMP) variant interpretation guidelines,^33^, ^34^, ^35^ and with prospects for gene‐specific therapies being developed, we performed detailed genotype–phenotype analyses of *ITPR1* missense variants. We describe the genetic variants and clinical features for a cohort of 46 patients with early‐onset ataxia, highlighting cases where cerebellar atrophy was demonstrated by serial imaging, and correlate the imaging findings with the clinical phenotypes. Genotype–phenotype correlation has previously been hindered by inconsistent transcript annotation in the literature.^18^, ^19^, ^20^, ^31^, ^36^ We investigated *ITPR1* expression by quantitative polymerase chain reaction (PCR) to determine relative transcript levels and standardized *ITPR1* variant nomenclature. Our data provide valuable information for the clinical interpretation of *ITPR1* missense variants and a focus for future preclinical studies towards new therapeutics.

## Methods

### The Deciphering Developmental Disorders Cohort

The Deciphering Developmental Disorders (DDD) study investigated children with undiagnosed developmental disorders across the UK and Ireland, utilizing exome sequencing in molecular diagnostics. Written informed consent for all patients was obtained through the DDD study,^37^, ^38^ or directly by their physician.

The DDD diagnostics framework has been described previously.^39^, ^40^ Briefly, fragmented genomic DNA was used for targeted pull‐down with a custom Agilent SureSelect 55 MB Exome Plus Enrichment System (Agilent, Santa Clara, CA, USA) and 75‐base pair paired‐end reads were sequenced on an Illumina HiSeq. Average sequencing depth (ratio of sequenced bases to targeted bases) was 903 across the whole targeted sequence or 933 across autosomal targets only. Alignment was performed with the Burrows–Wheeler Aligner (v.0.59), and realignment around indels was performed with the Genome Analysis Toolkit (GATK).^40^ Putative de novo variants were identified from exome data with DeNovoGear software.

The DDD study identified 62 patients with variants in *ITPR1*. We filtered these individuals according to: (1) absence of other pathogenic variants; (2) conservation of nucleotides and amino acids; (3) low allele frequency in ExAC and gnomAD; and (4) availability of clinical information. Variants were considered disease‐causing if they met the ACMG criteria for likely pathogenic or pathogenic^33^ and were associated with an appropriate phenotype. Five cases where a variant of unknown significance (VUS) was considered the most likely cause of the probands' phenotypes were included to increase the likelihood of determining pathogenicity in the future.

### Clinical Collaborations

Additional individuals were identified through specialist ataxia or neurogenetics clinics in Brazil, Finland, Ireland, Israel, Italy, Spain, and the UK. Targeted sequencing across a panel of known ataxia genes, or exome sequencing, was performed, and candidate variants were confirmed by Sanger sequencing. Sequencing, sequence analysis, variant calling, and variant annotation were performed according to in‐house protocols of the respective National Health Service (NHS) genetics laboratories or external laboratories.

All variants were analyzed by the variant interpretation programme Alamut (http://www.interactive-biosoftware.com), and by the standard pathogenicity prediction programmes Polyphen,^41^ SIFT,^42^ and CADD.^43^ Nucleotide conservation was estimated by PhyloP.^44^

### The 100,000 Genomes Cohort

The 100,000 Genomes Project, funded by NHS England, investigates patients with undiagnosed rare disease and/or cancer using genome sequencing. Details of the diagnostics pipeline are published.^45^ Briefly, genome sequencing was performed with TruSeq DNA PCR‐free sample preparation (Illumina) on a HiSeq 2500 sequencer with mean depth of 32× and a depth greater than 15× for at least 95% of the reference human genome. Genome sequencing reads were aligned to the Genome Reference Consortium human genome build 37 (GRCh37) with Isaac Genome Alignment Software. Family‐based variant calling of single‐nucleotide variants (SNVs) and indels was performed with Platypus variant caller. Variants were interpreted against the ACMG criteria.

### Total RNA Isolation and cDNA Synthesis

Total RNA from human fetal cerebellar tissue (female, 20 post‐conception weeks) was acquired commercially (AMS Biotechnology (Europe) Ltd., Abingdon, UK). Postnatal cerebellar samples were obtained from the Oxford Brain Bank (REC 15/SC/0639). Total RNA was isolated using the RNeasy Mini kit (QIAGEN Ltd., Manchester, UK) according to the manufacturer's instructions and eluted in nuclease‐free water. Approximately 30 mg of cortical cerebellar tissue was sonicated for 10 s using a Soniprep 150 Ultrasonic Disintegrator (MSE (UK) Ltd, London, UK) in RLT lysis buffer (QIAGEN). Samples were kept on ice during handling. 1000 ng of total RNA and oligo(dT) primers were used to synthesize cDNA with the SuperScript III First‐Strand Synthesis System (Thermo Fisher Scientific, Paisley, UK) according to the manufacturer's instructions. RNA concentrations were measured using a NanoDrop 2000 Spectrophotometer (Thermo Fisher Scientific).

### Quantitative PCR

Quantitative PCR (qPCR) was performed using the Fast SYBR Green Master Mix (Thermo Fisher Scientific) with a StepOnePlus Real‐Time PCR System (Applied Biosystems, Paisley, UK). Custom qPCR primers were designed to target a region shared by all *ITPR1* transcripts (exon–exon junction between exons 1 and 2) or alternatively spliced isoforms. A duplication event around the S1 site prevented the reliable quantification of the transcripts that lack S1 (ie, S1− transcripts). Thus, the S1+ transcripts are presented as a fraction of total *ITPR1* mRNA. For the S2 and S3 sites, we used one primer pair to quantify transcripts that include the alternatively spliced site (S2+ and S3+) and one primer pair spanning the junction of the flanking sequences (S2− and S3−). The data are presented as a fraction of S+ to S− transcripts. The qPCR primers are listed in Supplementary Table S1. Relative expression levels were normalized to *ACTINB* and *GAPDH* using the standard 2^−ΔΔCT^ technique.

### Analysis of Single‐Nucleus RNA Sequencing Data

The single‐nucleus RNA sequencing dataset used in this study was published previously.^11^ Re‐analysis was performed as in the original publication using Seurat^46^ and other necessary packages in R. Data on *ITPR1* in the developing human brain was extracted from the BrainSpan Developmental Transcriptome dataset (RNA‐Seq Genome v10).^47^, ^48^

## Results

### Novel *ITPR1* Variants and Standardized Variant Nomenclature

To characterize previously unreported cases of spinocerebellar ataxia type 29 (SCA29) and Gillespie syndrome (GLSP), we utilized next‐generation sequencing data from the DDD study (Table S2), the 100,000 Genomes Project, and multiple clinical collaborations (see Supplementary Fig. S1 for study flow). Overall, we identified 46 probands who met our filtering criteria with 52% of individuals carrying a known missense variant (Table 1). Some 52% (24/46) of cases were de novo and 82% (23/28) of variants had been classified as disease‐causing by the reporting laboratories (Table 1). The molecular characteristics of each variant are shown in Supplementary Tables S3 (DDD, clinical collaborations) and S4 (100,000 Genomes Project).

**TABLE 1:** Summary table of *ITPR1* variants in the present study

Parameter	Percentage % (n)
Variant^a^	
Known	52% (24/46)
Novel	48% (22/46)
Variant origin	
De novo	52% (24/46)
Inherited	9% (4/46)
Unknown	39% (18/46)
Variant classification	
Pathogenic	36% (10/28)
Likely pathogenic	46% (13/28)
Variant of unknown significance (VUS)	18% (5/28)

^a^ For each individual; range of individuals with the same variant, 1–7.

**FIG. 1:**
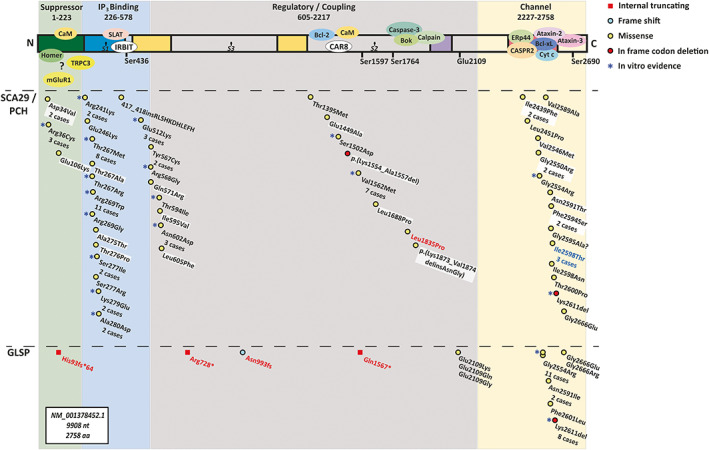
Pathogenic IP_3_R1 missense variants cluster in three functional domains. The *ITPR1* gene encodes a protein of 2758 residues with four functional domains (residues 1–223: suppressor [green], residues 226–578: IP_3_‐binding [blue], residues 605–2217: regulatory [gray], residues 2227–2758: transmembrane channel [yellow]), and multiple interaction partners. Shown are previously published variants and variants identified as part of the present study, categorized as internal truncating (red square), frameshift (blue circle), missense (yellow circle), and in‐frame codon deletion (red circle), and grouped by diagnosis (SCA29/pontocerebellar hypoplasia [PCH], and GLSP). Homozygous variants are shown in red text, whereas novel variants are highlighted on a white background. Variants associated particularly with PCH are shown in blue text. Variants with published experimental validation are further denoted by a blue star. Each variant is listed with the known number of cases per variant indicating multiple mutational hotspots.

The 46 probands carried 28 unique *ITPR1* variants, of which 17 had not been reported previously and were distributed in the N‐terminus (n = 8), the regulatory domain (n = 4), and the C‐terminus (n = 5) (Fig. 1, Supplementary Tables S3 and S4). The first 200 residues in the N‐terminus comprise the suppressor domain,^4^ containing a single known pathogenic variant (p.Arg36Cys) with a gain‐of‐function effect of increased IP_3_ binding.^49^ As part of our study, we identified two cases harboring the same p.Arg36Cys variant (one published recently with limited phenotypic information^50^) and three cases of previously unreported variants p.Asp34Val and p.Glu106Lys. The aspartate and arginine at positions 34 and 36, respectively, have been shown to regulate the function of the suppressor domain.^49^, ^51^ We therefore confirmed the p.Arg36Cys as a key suppressor domain variant, potentially destabilizing the inhibitory effect this domain has on IP_3_ binding.^4^ A review of the literature and our data further highlighted variants p.Thr267Met, p.Arg269Trp, p.Val1562Met, p.Gly2554Arg, and p.Lys2611del as mutational hotspots (Fig. 1). In our entire dataset, only one case, harboring the p.Val1562Met variant, was not associated with ataxia but global development delay (Supplementary Table S4). This variant lies within the CAR8‐binding region and was the first published missense variant associated with SCA29 of a very mild phenotype.^52^ Finally, in our cohort, the only variants associated with GLSP were p.Gly2554Arg and p.Lys2611del, both previously reported in GLSP.^20^

Variant calling for the present study revealed significant inconsistencies in *ITPR1* variant nomenclature which confounded variant interpretation. The variable terminology is explained by alternative splicing of the *ITPR1* mRNA at three sites (S1, S2, and S3, corresponding to exons 12, 40–42, and 23, respectively, Supplementary Fig. S2),^53^, ^54^, ^55^ resulting in at least three different mRNA transcripts being used for variant mapping.^18^, ^19^, ^20^, ^31^, ^36^ Based on previously published RNA sequencing datasets,^11^, ^12^, ^47^, ^48^ *ITPR1* mRNA is expressed in cerebellar Purkinje cells from early embryonic development to adulthood (Supplementary Fig. S3). Using custom qPCR primers (Supplementary Fig. S2A), we determined that the three splice sites are also expressed from fetal to postnatal samples (Supplementary Fig. S2B–D). Concurring with previous data in rodents,^54^ the S3 site undergoes a shift towards shorter isoforms whereby the S3− transcripts predominated in the postnatal samples (Supplementary Fig. S2D). Taken together, our data suggest that the three alternatively spliced sites are expressed in the human cerebellum. Consequently, we mapped disease‐causing variants, both from published literature (Supplementary Table S5) and our own datasets (Supplementary Tables S3 and S4) to the longest *ITPR1* transcript (NM_001378452.1) (Fig. 1), which contains all three splice sites. This protein isoform is 2758 amino acid residues in length and is listed as the canonical IP_3_R1 isoform in UniProt (Q14643‐1) and Ensembl (ENST00000649015.2, MANE select transcript).

### Core Phenotypes, Neuroradiological Findings, and Atypical Features

Detailed clinical information is summarized in Table 2. Most cases (n = 40, 87%) resemble SCA29 clinically with five cases of aniridia resulting in the diagnosis of GLSP. Only one individual presented with adult‐onset ataxia requiring further evaluation of variant pathogenicity (p.Glu1449Ala, VUS). Some 92% of cases with sufficient data were identified within the first year of life with initial symptoms consisting of hypotonia, delayed developmental milestones, or ophthalmological findings (Table 2). Independent sitting was delayed until after the first year of life in 67% of individuals, and a significant proportion of individuals had not attained independent walking at last assessment (Table 2). Ataxia was observed in 97% of individuals for whom clinicians had specifically reported presence or absence of ataxia. Intellectual disability was formally diagnosed in 45% of cases. Considering the high proportion of cases with milder cognitive impairments (ie, learning disability, Supplementary Tables S6 and S7), the core phenotype of SCA29 is ataxia with cognitive impairment (Table 2). Nevertheless, our dataset does include individuals with normal cognition (5/34, 13.9%) or limited motor symptoms (4/28, 14.3%), which is in agreement with the range of phenotypes described in published case reports of *ITPR1* missense variants (n = 86, Supplementary Tables S5 and S8). Three patients received a genetic diagnosis of SCA29 as part of this study.

**TABLE 2:** Summary table of phenotypic findings in the present study

Parameter	Percentage % (n)
Age at onset	
Congenital	48% (13/27)
0–1 y	44% (12/27)
1–5 y	8% (2/27)
Symptom at presentation	
Hypotonia	36% (9/25)
Developmental delay	48% (12/25)
Ophthalmological finding	36% (9/25)
Ataxia	12% (3/25)
Delayed motor milestones	
Independent sitting >1 y	67% (14/21)
Independent walking >2 y	23% (6/26)
Independent walking not attained	50% (13/26)
Developmental delays	
Motor	93% (26/28)
Speech	76% (19/25)
Global	60% (21/35)
Intellectual disability	45% (14/31)
Cerebellar symptoms	
Hypotonia	87% (27/31)
Ataxia	97% (34/35)
Dysmetria	71% (17/24)
Tremor	67% (16/24)
Eye phenotype	
Normal	29% (10/35)
Aniridia^a^	14% (5/35)
Nystagmus	31% (11/35)
Strabismus	9% (3/35)
Ptosis	11% (4/35)

^a^ Aniridia, iris hypoplasia, or a large, non‐reactive pupil with irido‐lenticular straining.

Atypical features and neuroradiological findings are presented in Table 3 and Supplementary Tables S9 and S10. The neuroradiological findings were heterogenous (Table 3, Supplementary Tables S9 and S10). In our dataset, brain imaging of cases was variously reported as unremarkable (28%), cerebellar hypoplasia (24%), or cerebellar atrophy (62%), and occasionally both hypoplasia and atrophy. In those cases in which serial scanning was available (see variants p.Arg269Trp, p.Leu605Phe, p.Leu1688Pro, p.Ile2439Phe, p.Gly2554Arg, p.Phe2594Ser, Supplementary Tables S9 and S10), the degree of atrophy did not correlate with the subjective severity of ataxia or intellectual disability, both of which commonly remain stable or may improve. Importantly, in none of the cases with proven cerebellar atrophy was a decline in function or clinical regression reported. Extra‐neurological features were reported in several patients without genotype–phenotype association (Table 3, Supplementary Tables S9 and S10). These range from dysmorphic features in the face or extremities to abnormalities in other organ systems such as the heart, where atrioventricular septal defects and pulmonary stenosis are seen in 9% (3/35) and 6% (2/35) of individuals with available data. The individual with adult‐onset ataxia (p.Glu1449Ala, VUS) presented with distal sensory neuropathy, which has not previously been reported with *ITPR1* variants.

**TABLE 3:** Summary of neuroimaging and extra‐cerebellar findings in the present study

Parameter	Percentage % (n)
Neuroimaging	
Unremarkable	28% (8/29)
Cerebellar hypoplasia	24% (7/29)
Cerebellar atrophy	62% (18/29)
Cardiovascular abnormalities	
Septal defects	9% (3/35)
Pulmonic stenosis	6% (2/35)
Musculoskeletal abnormalities	
Microcephaly	11% (4/35)
Scoliosis	3% (1/35)
Facial findings	23% (8/35)
Extremities	17% (6/35)
Seizures	9% (3/35)

### Pathogenic Missense Variants Cluster in Three Functional Domains

To evaluate genotype–phenotype correlations in the three‐dimensional space, we projected published missense variants onto the rat IP_3_R1 tetrameric protein structure.^9^ Homozygous truncating variants (p.His93fs*64, p.Arg728*, and p.Gln1567*) are only reported in GLSP.^19^, ^56^, ^57^ Known missense variants cluster in two major groups: an N‐terminal cluster consisting solely of SCA29‐associated missense variants (Supplementary Fig. S4A) and a C‐terminal cluster in the channel domain containing both SCA29 and GLSP variants (Supplementary Fig. S4B). However, there were no obvious genotype–phenotype correlations: for example, the C‐terminal p.Gly2554Arg, p.Lys2611del, and p.Gly2666Glu variants are mainly associated with GLSP, but there are recent reports of single individuals carrying these variants without aniridia.^31^, ^58^ The N‐ and C‐terminal clusters correlate well with regions of relatively low missense variant density in gnomAD (Supplementary Fig. S5A), indicating that these regions are sensitive to missense variation, whereas the lowest regional missense variant constraint (ie, highest tolerance to missense variation) in ExAC is found in the last third of the regulatory domain (Supplementary Fig. S5B).^59^

Despite the lower missense constraint in the regulatory domain, three *ITPR1* variants have previously been identified in the CAR8‐binding region within the regulatory domain,^31^, ^60^ at least two of which impair IP_3_R1‐CAR8 interaction, thereby releasing IP_3_R1 from CAR8‐mediated inhibition.^7^ Our dataset includes nine individuals with a variant located in the CAR8‐binding region (p.Glu1449Ala (VUS, n = 1), p.Lys1554_Ala1557del (likely pathogenic, n = 1), p.Val1562Met (likely pathogenic, n = 5), p.Leu1688Pro (likely pathogenic, n = 1), and p.Lys1873_Val1874delinsAsnGly (VUS, n = 1)) (Fig. 1), pointing to the CAR8‐binding region as an additional cluster of variants. Thus, disease‐causing variants in *ITPR1* group in three regions: the N‐terminus (49% of variants, 47% of cases), the CAR8‐binding region in the regulatory domain (16% of variants, 13% of cases), and the C‐terminal channel domain (35% of variants, 40% of cases) (Fig. 1).

Finally, to ensure that our data were unbiased towards predefined ACMG guidelines, we mapped the *ITPR1* SNVs in the 100,000 Genomes data that were excluded as benign. The 100,000 Genomes dataset contained 225 probands with 193 unique *ITPR1* variants of which 11% were predicted to be deleterious to protein function by Polyphen^41^ and Sift^42^; variants were more frequently classified as deleterious by Polyphen than Sift (29% vs. 13%). Using Human Phenotype Ontology terms, we further narrowed down the number of cases to focus on neurological phenotypes (ataxia [n = 27], global developmental delay [n = 39], and intellectual disability [n = 2]). The suppressor, IP_3_‐binding, and channel domains contained 2–3 excluded variants each, whereas 30 variants were found in the regulatory domain (Supplementary Fig. S6). When normalized to the number of amino acid residues for each domain, the number of these variants in the regulatory domain is 2–14‐fold higher. The data confirm the higher missense tolerance observed for the regulatory domain, and that combined with the clinical phenotypes, variant frequency, and prediction algorithms in each case, these variants are unlikely to be pathogenic (Supplementary Fig. S5B).

## Discussion

To our knowledge, this dataset represents the largest cohort of patients with missense variants in *ITPR1*. The data show that these variants cluster around three functional domains of IP_3_R1. Two have been previously described: the IP_3_‐binding domain and the C‐terminal channel domain. In addition, we found an enrichment of variants with the core phenotype in the CAR8‐binding region. Variants outside these clusters should be interpreted with considerable caution. We have defined the core phenotype of SCA29 and GLSP as ataxia with cognitive impairment and hypothesize that cerebellar atrophy does not correspond with symptom progression. Finally, we have standardized *ITPR1* variant nomenclature to the longest transcript (NM_001378452), enabling easier cross‐comparison between datasets. Overall, these results have an important bearing on variant interpretation in clinical practice as well as the pursuit for new therapeutics.

**FIG. 2:**
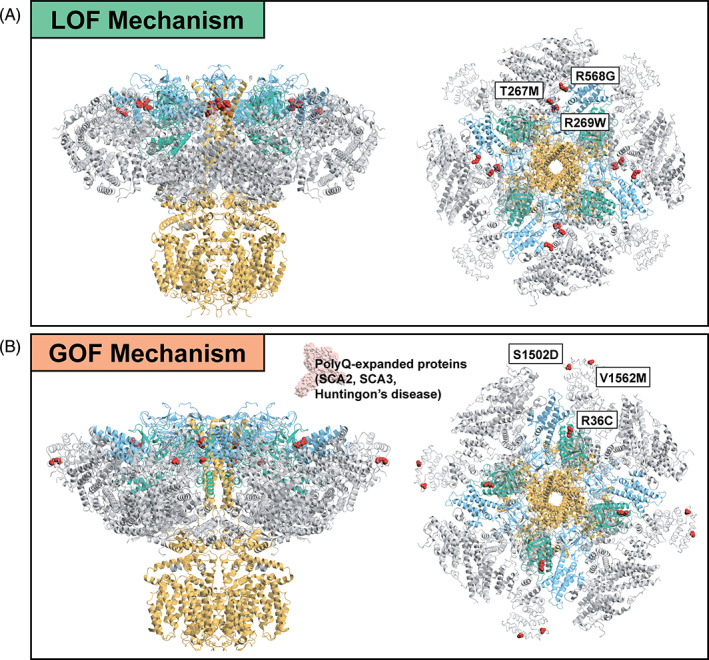
Different functional clusters of IP_3_R1 missense variants guide therapeutic design. (**A**) Loss‐of‐function (LOF) variants such as p.Thr267Met, p.Arg269Trp, and p.Arg568Gly are likely to require potentiators of IP_3_R1 channel function to be targeted therapeutically. (**B**) Gain‐of‐function (GOF) variants p.Arg36Cys, p.Val1562Met, and p.Ser1502Asp interfere with different suppression mechanisms of the IP_3_R1 channel potentially requiring IP_3_R1 inhibitors as therapeutics. Polyglutamine‐expansion disorders (ie, SCA2, SCA3, Huntington's disease, and familial Alzheimer's disease) involving a dysregulated IP_3_R1 channel may also respond to IP_3_R1 inhibition. The figure shows the protein structure for the rat IP_3_R1 (Protein Data Bank: 7LHF) and ataxin‐3 (light pink, Protein Data Bank: 3O65).

The distribution of variants across different domains of the IP_3_R1 protein suggests both loss‐of‐function (LOF) and gain‐of‐function (GOF) disease mechanisms (Fig. 2) with important implications for therapeutic development. The majority of SCA29 and GLSP cases harbor variants in the IP_3_‐binding and channel domains. In the IP_3_‐binding domain, hotspot variants p.Thr267Met, p.Arg269Trp and others (ie, Tyr567, Arg568) directly impact hydrogen bond formation between IP_3_ and its binding domain,^3^, ^61^ significantly reducing IP_3_‐binding affinities and leading to dominant negative effects. These variants may respond to positive modulators of IP_3_R1 channel function. Interestingly, the first IP_3_R1 potentiator was recently published with evidence to suggest it may reverse the LOF effect of the p.Thr267Met and p.Arg269Trp variants.^62^ In contrast, variants p.Arg36Cys, p.Val1562Met, and p.Ser1502Asp confer a GOF effect by interfering with different suppression mechanisms of IP_3_R1 channel function (ie, destabilizing the suppressor domain and the interaction with CAR8).^7^, ^49^ Although these variants are rare in our dataset, a similar GOF effect is seen in other spinocerebellar ataxias (SCA2, SCA3), Huntington's disease, familial Alzheimer's disease, and a recent mouse model of IP_3_R1 function, again having therapeutic implications.^23^, ^24^, ^25^, ^26^, ^28^, ^29^, ^63^ Thus, an IP_3_R1 inhibitor may provide a plausible therapeutic option to target a range of disorders involving a dysregulated IP_3_R1 channel.

Our SCA29/GLSP cohort reveals variable phenotypic expressivity without correlation with the genotype of the individual, a feature seen in several non‐repeat expansion ataxias.^50^ However, our data are the first to evaluate a large cohort of *ITPR1* missense variants alone. This is of relevance, as deletions in *ITPR1* cause SCA15 with a very different phenotype and underlying mechanistic basis from SCA29.^50^ Currently, early‐onset *ITPR1*‐associated ataxias are diagnosed as GLSP distinguished by aniridia^19^, ^20^ or SCA29^18^ with a few cases of severe pontine/cerebellar hypoplasia.^21^ N‐Terminal variants are solely associated with SCA29, while C‐terminal variants can cause either GLSP (p.Gly2554Arg and p.Lys2611del) or SCA29. Some evidence points to a downstream transcription initiation site (TSS) 5′ to exon 57 of the *ITPR1* gene giving rise to the aniridia in GLSP,^64^ but this TSS does not account for variants that have been associated with both GLSP and SCA29,^31^, ^58^ or homozygous N‐terminal truncating variants associated with GLSP.^19^, ^56^, ^57^ Even within the SCA29 diagnosis,^18^, ^52^ the phenotype can range from mild learning disabilities without ataxia to severe, debilitating ataxia and significant intellectual disability. Therefore, the continuum of symptoms is unexplained by current genotype–phenotype evidence and points to the involvement of additional modulators. Nevertheless, the severity of symptoms in SCA29 emphasizes the necessity for early diagnosis and targeted rehabilitation.

Our data highlight two features of SCA29/GLSP that are less characterized: the distinction between cerebellar hypoplasia and atrophy, and extra‐neurological features. Available evidence suggests that the natural history of SCA29 and GLSP is non‐progressive. However, recent publications have provided evidence that superior vermian and/or hemispheric cerebellar atrophy may represent a hallmark of *ITPR1*‐related disorders.^32^, ^65^ We report on at least six individuals in whom cerebellar hypoplasia was excluded by early brain imaging (3 months to 2 years) but who later developed cerebellar atrophy without evidence of clinical regression. At least four individuals were shown to have cerebellar hypoplasia shortly after birth. This distinction between hypoplasia and atrophy is important, as atrophy usually implies neurodegeneration and is unexpected in patients with clinically non‐progressive disease. Repeated imaging can distinguish between the two, but we hypothesize that the clinical significance and mechanistic basis of cerebellar atrophy is uncertain. A prospective natural history study of SCA29 utilizing serial scanning and objective disease severity measures is necessary to confirm this finding. Such a study should further aim to provide systematic data on prevalence of *ITPR1*‐related diseases, and to add genomic diversity by including individuals from underrepresented populations.

Several individuals in the cohort present with structural malformations (eg, dysmorphic features and congenital heart defects). Previously, *ITPR1* variants have been linked to hemifacial microsomia^66^ and cardiovascular malformations,^57^ mainly in association with GLSP. Interestingly, atrial septal defects and other cardiac abnormalities are seen in at least seven published cases and five individuals in our cohort suggesting a higher‐than‐expected prevalence considering the size of the cohort and the prevalence of congenital heart disease in the general population (1.4 and 3.1 per 1000 live births for atrial and ventricular defects, respectively).^67^ IP_3_R1 and IP_3_R2 have been implicated in the perturbation of cardiogenesis via deficient calcineurin‐NFATc signaling,^68^ but causation between dysfunctional IP_3_R1 and congenital heart disease remains unclear.

In conclusion, our data provide evidence for a core SCA29/GLSP phenotype resulting from pathogenic IP_3_R1 missense variants in specific protein domains. We have standardized *ITPR1* variant nomenclature to enable easier cross‐comparison of variant novelty and pathogenicity between datasets. Our data highlight a diverse expression of *ITPR1* transcripts in the cerebellum raising the question whether alternative splicing of *ITPR1* mRNA could help explain why *ITPR1* haploinsufficiency (SCA15) only manifests in adulthood. Finally, we identify key IP_3_R1 variants for preclinical research to guide therapeutic design for SCA29/GLSP and other disorders involving a dysregulated IP_3_R1 channel.

## Financial Disclosures of All Authors for the Preceding 12 Months

This project has received funding from the European Union's Horizon 2020 research and innovation programme under the Marie Sklodowska‐Curie Grant Agreement No. 101023312 PEDIATAX (J.P.T). J.P.T. was further supported by the Research Council of Finland (Grant No. 356676), the Sigrid Jusélius Foundation, the Orion Research Foundation sr, and the Alma and K.A. Snellman Foundation, Oulu. Finland. This publication arises from research funded by the John Fell Oxford University Press Research Fund (J.P.T.). D.S. is funded by MRC. G.K.T. is supported by a MRC Senior Clinical Fellowship (MR/V007068/1). S.E. serves on the board of DECIPHER and received royalties for Emery's Elements of Medical Genetics. C.S.‐S. has received reimbursements for expert testimonies in medicolegal case reports. K.G. is supported by the Alma and K.A. Snellman Foundation, Oulu, Finland, and the Foundation for Paediatric Research. J.U. serves on the advisory board of Pfizer (DMD Advisory Board, Finland) and is funded by the Research Council of Finland (Decision No. 331436), the Foundation for Paediatric Research (Finland), and a state subsidy of the Oulu University Hospital, Finland. A.J.M.B. is supported by a Wellcome PhD Training Fellowship for Clinicians and the 4Ward North PhD Programme for Health Professionals (223521/Z/21/Z). S.A.L. serves on the advisory boards of the European Union of Medical Specialists, the European Society of Human Genetics, and the International Rare Disease Research Consortium. S.A.L. is employed by the HSE Ireland and has been funded by the Adelaide Health Foundation Ireland. A.P.M. has received speaker fees and royalties from Ipsen UK for the Livechart Botulinum Toxin Treatment Outcome system, and royalties for the ‘Handbook of Botulinum Toxin Treatment’ (Blackwell Science). W.D.J. is an honorary associate professor at University College London, London, and a patron of the ‘National Wiedemann–Steiner Warriors’ charity. E.B.E.B. is funded by the UKRI‐MRC, UKRI‐BBSRC, Cancer Research UK, LifeArc, and John Fell OUP Research Fund. A.H.N. was funded by Ataxia UK, John Fell OUP Research Fund, Henry Smith Charity, Nuffield Department of Clinical Neurosciences (University of Oxford), and Action Medical Research (GN2063). Authors declare employment by their respective institutes.

## Author Roles

Conceptualization: J.P.T., E.B.E.B., A.H.N.

Methodology: J.P.T., R.P.S., S.M., D.S.

Software: J.P.T., R.P.S., S.M., D.S.

Validation: R.P.S.

Formal analysis: J.P.T., R.P.S., A.H.N.

Investigation: J.P.T., R.P.S., S.M.

Resources: J.P.T., S.M., S.D., M.M., F.E., D.S., H.S., G.K.T., T.D., P.J.M., D.J., M.H., S.E., C.S.‐S., A.Z., A.D., M.S., A.S., R.E.H., G.J., H.H., G.C., J.J., J.W., M.E.S., P.C., J.R., L.B., T.L.‐S., P.P., S.R., K.G., J.U., H.C., E.M., S.J., A.J.M.B., K.M., H.K., M.B., R.K., S.A.L., I.M.A., A.P.M., W.D.J., A.H.N.

Data curation: J.P.T., R.P.S., S.M., D.S.

Writing – original draft: J.P.T., A.H.N.

Writing – review and editing: J.P.T., R.P.S., S.M., S.D., M.M., F.E., D.S., H.S., G.K.T., T.D., P.J.M., D.J., M.H., S.E., C.S.‐S., A.Z., A.D., M.S., A.S., R.E.H., G.J., H.H., G.C., J.J., J.W., M.E.S., P.C., J.R., L.B., T.L.‐S., P.P., S.R., K.G., J.U., H.C., E.M., S.J., A.J.M.B., K.M., H.K., M.B., R.K., S.A.L., I.M.A., A.P.M., W.D.J., E.B.E.B., A.H.N.

Visualization: J.P.T., R.P.S.

Supervision: E.B.E.B., A.H.N.

Project administration: E.B.E.B., A.H.N.

Funding acquisition: J.P.T., E.B.E.B., A.H.N.

## Supporting information


**FIG. S1.** Study flow. Individuals from three clinical cohorts were filtered (Deciphering Developmental Disorders and 100,000 Genomes cohorts) and interpreted against the American College of Medical Genetics and Genomics (ACMG) criteria. A total of 46 cases and 28 unique variants (ie, four overlapping variants: T267A, R269W, V1562M, G2554R) were retained in the final analyses.
**FIG. S2.** Alternative splicing of *ITPR1* transcripts in the fetal and postnatal cerebellum. (**A**) Quantitative PCR primers were designed to amplify the three alternatively spliced regions of the *ITPR1* mRNA at S1, S2, and S3, with one primer pair targeting transcripts that contain the alternatively spliced site (S+), while one primer pair spans the junction of the flanking sequences (S–). Exon–exon junction at the end of exon 23 (S3+) is underscored. (**B‐D**) Relative expression data were normalized to *ACTINB* and *GAPDH*. (**B**) The S1+ mRNA levels presented as a fraction of total *ITPR1* expression is stable across the time‐course (n = 3). (**C**) The S2– and S2+ transcripts are expressed at an approximate 1:1 ratio across the time‐course (n = 3). (**D**) Splicing at the S3 site appears to undergo a shift towards shorter isoforms whereby the S3– transcripts predominate in the postnatal samples (n = 3). *m* = male, *f* = female.
**FIG. S3.** Distribution of *ITPR1* mRNA expression across development and adulthood. (**A**) Expression of *ITPR1* mRNA is enriched in the developing Purkinje cell population in a recently published single‐nuclei RNA sequencing dataset (Aldinger et al., 2021). (**B**) A heatmap showing the expression of *ITPR1* mRNA in the Purkinje cell subset from 9 PCW to 20 PCW in the developing cerebellum (Aldinger et al., 2021). Expression data were scaled per gene (mean expression (± variance)) across samples. (**C**) *ITPR1* mRNA levels further increase postnatally in the BrainSpan atlas of the developing human brain dataset.
**FIG. S4.** Projecting previously published missense variants on to the IP_3_R1 protein structure. Published disease‐causing variants in *ITPR1* were projected onto the four subunits of a recently resolved IP_3_R1 protein structure in a lipid bilayer (PDB: 7LHF). Protein modeling was performed using PyMOL (version 2.6.0a0). (**A**) The distribution of all published *ITPR1* variants displays two clusters in the N‐ and C‐terminal domains: N‐terminal suppressor domain (green) and IP_3_ binding core (blue), and C‐terminal channel domain (yellow). (**B**) The C‐terminal disease‐causing variants were categorized into four groups: all C‐terminal variants; variants associated with both SCA29 and Gillespie syndrome (GLSP) (red); and variants that cause either SCA29 (red and pink) or GLSP (red and blue). No apparent genotype–phenotype correlations are observed.
**FIG. S5.** Benign missense variants across the *ITPR1* gene. (**A**) All variants in gnomAD, presumably benign, are shown along the *ITPR1* gene. Note the relative paucity of variants towards the 5′ and 3′ ends in comparison to the regulatory domain. (**B**) The regional missense constraint track shows the Observed/Expected ratio for three regions of the *ITPR1* gene with lower‐than‐expected frequency of missense variants for parts of the regulatory domain. The highest density of missense variants in gnomAD correlates with the lowest regional missense constraint (highest tolerance) found for the *ITPR1* gene in the ExAc data (Samocha et al., 2017).
**FIG. S6.** Excluded single‐nucleotide variants (SNVs) from the 100,000 Genomes database cluster in the regulatory domain. The 100,000 Genomes dataset contained 225 probands with 193 unique *ITPR1* SNVs. Using human phenotype ontology (HPO) terms, the variants were further narrowed down by association with neurological phenotypes such as ataxias (27 patients), global developmental delay (39 patients), and intellectual disability (2 patients). Overall, there were 37 excluded SNVs based on inappropriate phenotypes (gray sphere), frequency in variant databases (ExAC, gnomAD) (blue sphere), and benign classification by prediction algorithms (red sphere). The number of excluded SNVs was 2–14‐fold higher in the regulatory domain than the other domains when normalized to the number of amino acid residues.

**TABLE S1.** Primers used for quantitative polymerase chain reaction (PCR).
**TABLE S2.** DECIPHER IDs included in the dataset.
**TABLE S3.** Molecular characteristics of *ITPR1* variants in the Deciphering Developmental Disorders and clinical collaborations cohort.
**TABLE S4.** Molecular characteristics of *ITPR1* variants in the 100,000 Genomes database.
**TABLE S5.** Known disease‐causing *ITPR1* variants excluding large deletions mapped to the four main IP_3_R1 protein isoforms.
**TABLE S6.** Proband phenotypes in the Deciphering Developmental Disorders and clinical collaborations cohort.
**TABLE S7.** Proband phenotypes in the 100,000 Genomes cohort.
**TABLE S8.** Range of phenotypes associated with published disease‐causing variants in *ITPR1*.
**TABLE S9.** Structural abnormalities and imaging data in the Deciphering Developmental Disorders and clinical collaborations cohort.
**TABLE S10.** Structural abnormalities and imaging data in the 100,000 Genomes cohort.

## Data Availability

The data that support the findings of this study are available on request from the corresponding author. The data are not publicly available due to privacy or ethical restrictions.
